# A Morphological Study of Distal Femoral Varus Deformity

**DOI:** 10.7759/cureus.64822

**Published:** 2024-07-18

**Authors:** Junya Hara, Akira Maeyama, Tetsuro Ishimatsu, Taiki Matsunaga, Shizuhide Nakayama, Takuaki Yamamoto

**Affiliations:** 1 Department of Orthopaedic Surgery, Fukuoka University Faculty of Medicine, Fukuoka, JPN

**Keywords:** undercorrection, owhto, ldfa, distal femoral varus deformity, around knee osteotomy, total knee arthroplasty

## Abstract

Background

In the management of medial compartment knee osteoarthritis via around-knee osteotomy (AKO) and total knee arthroplasty (TKA), evaluating the lateral distal femoral angle (LDFA) is crucial. This angle reflects the presence of distal femoral varus deformity. This study aims to explore the relationship between LDFA and lower extremity bone morphology and identify factors contributing to a high LDFA.

Methods

A retrospective analysis was conducted on 59 patients who underwent AKO or TKA at our hospital. Alignment of the lower extremity was assessed using X-rays, and bone morphology was investigated through computed tomography (CT) employing the ZedKnee^®^ system^ ^(LEXI, Tokyo, Japan). Each measured parameter was analyzed.

Results

Our findings indicate a significant correlation between LDFA and several parameters, including age, femoral tibial angle (FTA), hip knee ankle angle (HKA), percentage of mechanical axis (% MA), femoral bowing angle, femoral tibial joint torsion, and the height of lateral and medial femoral condyles. A multiple-regression analysis determined that the most significant influences on LDFA were the heights of the femoral condyle, age, and HKA.

Conclusion

LDFA is significantly affected by the heights of the medial and lateral femoral condyles and tends to increase with age, possibly as a result of attrition of the medial femoral condyle. Given its significance, LDFA should be carefully considered as a preoperative indicator in AKO and TKA to guide surgical caution when LDFA is elevated.

## Introduction

Around-knee osteotomy (AKO) and total knee arthroplasty (TKA) are established surgical interventions for treating medial compartment knee osteoarthritis (OA) [1-6]. Evaluating the alignment of the lower extremity in the coronal plane, which is a critical aspect of both procedures, has been extensively studied [7-9].

In TKA, the mechanical alignment (MA) method, which involves performing osteotomies perpendicular to the functional axis of the femur and tibia, has traditionally been the gold standard [10,11]. However, kinematic alignment (KA) has recently emerged as an alternative, emphasizing the restoration of the patient's native constitutional alignment [12-14]. Accurate assessment of coronal plane alignment is crucial for the KA method, leading to the development of various assessment techniques and classifications [7-9]. The newly introduced Coronal Plane Alignment of the Knee (CPAK) classification [7] employs the lateral distal femoral angle (LDFA) and medial proximal tibial angle (MPTA) to classify knee alignment into nine distinct categories. This system helps predict the constitutional alignment of the patient, with LDFA and MPTA serving as critical metrics.

In AKO, several techniques, such as open wedge high tibial osteotomy (OWHTO), closed wedge high tibial osteotomy (CWHTO), and double level osteotomy (DLO) have been developed [1-3,15]. Technique selection is informed by the correction angle and various morphological parameters. For severe varus deformities requiring substantial correction, OWHTO may lead to an unnatural joint plane tilt, making DLO a preferable option as it corrects both femoral and tibial angles [16]. The LDFA and MPTA are important in determining the appropriate surgical approach. Furthermore, it has been observed that a pre-existing distal femoral varus deformity, indicated by a large LDFA, may lead to inadequate correction following the osteotomy [17-19].

Previous studies have indicated that LDFA may increase as a result of femoral bowing associated with increased varus deformity of the lower limb [20]. However, these studies typically only assessed X-rays and did not analyze three-dimensional bone morphology. Furthermore, although it is commonly reported that a large preoperative LDFA can lead to a loss of correction post-osteotomy, the morphological implications of a “large LDFA” have not been thoroughly examined. The purpose of this study was to investigate the relationship between mechanical LDFA and bone morphology by analyzing the lower extremity bone structure using 3DCT. It was hypothesized that changes in distal femoral morphology could affect the LDFA.

## Materials and methods

Patients

This study was approved by the Institutional Review Board of Fukuoka University Hospital (U21-11-010). A total of 86 patients who underwent AKO or TKA for medial compartment knee OA at our hospital from June 2021 to May 2022 were retrospectively studied. A total of 59 patients with 59 knees were included after excluding incomplete data (13 patients), rheumatoid disease (6 patients), and patients with a history of lower extremity surgery (8 patients). The maximum flexion contracture observed among the patients was minus 15 degrees in extension (2 patients).

Radiographic evaluation

**Figure 1:**
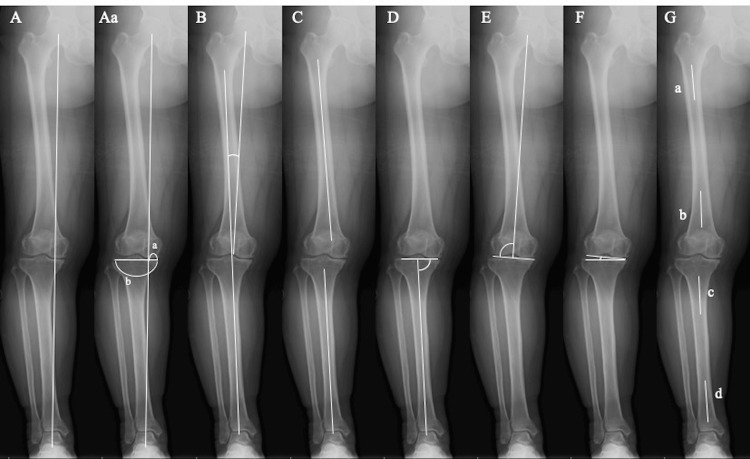
X-ray measurement parameters. X-ray evaluation was performed using standing anteroposterior one-leg standing full-length radiographs of the lower limb. (A) Mechanical axis (Aa) The % MA is calculated as the ratio of the horizontal distance from the medial edge of the tibial plateau to the intersection of the MA. % MA = a/b. (B) HKA: Angle between the femoral and tibial functional axes. (C) FTA: Angle between the femoral and tibial anatomical axes. (D) MPTA: Angle between the tibial anatomical axis and the joint line of the proximal tibia. (E) LDFA: Angle between the femoral functional axis and the distal femoral joint line. (F) JLCA: Angle between the distal femoral and proximal tibial joint line. (G) FBA: Angle between the proximal 1/4 line through the center of the femoral diaphysis and the distal 1/4 line. TBA: Angle between the proximal 1/3 line through the center of the tibial diaphysis and the distal 1/3 line.

X-ray evaluation involved acquiring standing anteroposterior full-length radiographs of the lower limb in a one-leg standing position to measure several key parameters: the percentage of mechanical axis (% MA), hip knee ankle angle (HKA), femoral tibial angle (FTA), medial proximal tibial angle (MPTA), lateral femoral angle (LDFA), joint line convergence angle (JLCA), femoral bowing angle (FBA), and tibial bowing angle (TBA) (Figure 1).

The MA is identified where the Mikulicz line (a line from the center of the femoral head to the center of the talus) crosses the most medial aspect of the tibial plateau. The % MA is calculated as the ratio of the horizontal distance from the medial edge of the tibial plateau to where it intersects the MA. The HKA angle measures the alignment between the femoral and tibial functional axes, where positive values indicate a varus deformity. The FTA represents the lateral angle between the femoral and tibial anatomical axes. The MPTA and the LDFA, respectively, define the medial and lateral angles between the tibial and femoral anatomical axes and their corresponding joint lines. The JLCA is the angle formed at the intersection of the distal femoral and proximal tibial joint lines, with positive values indicating a medial vertex. The FBA and TBA are defined by the angles between the lines drawn through the proximal and distal quarters of the femoral diaphysis, and the proximal and distal thirds of the tibial diaphysis, respectively [21,22]. Positive values for FBA and TBA suggest the presence of varus deformation.

CT evaluation

**Figure 2:**
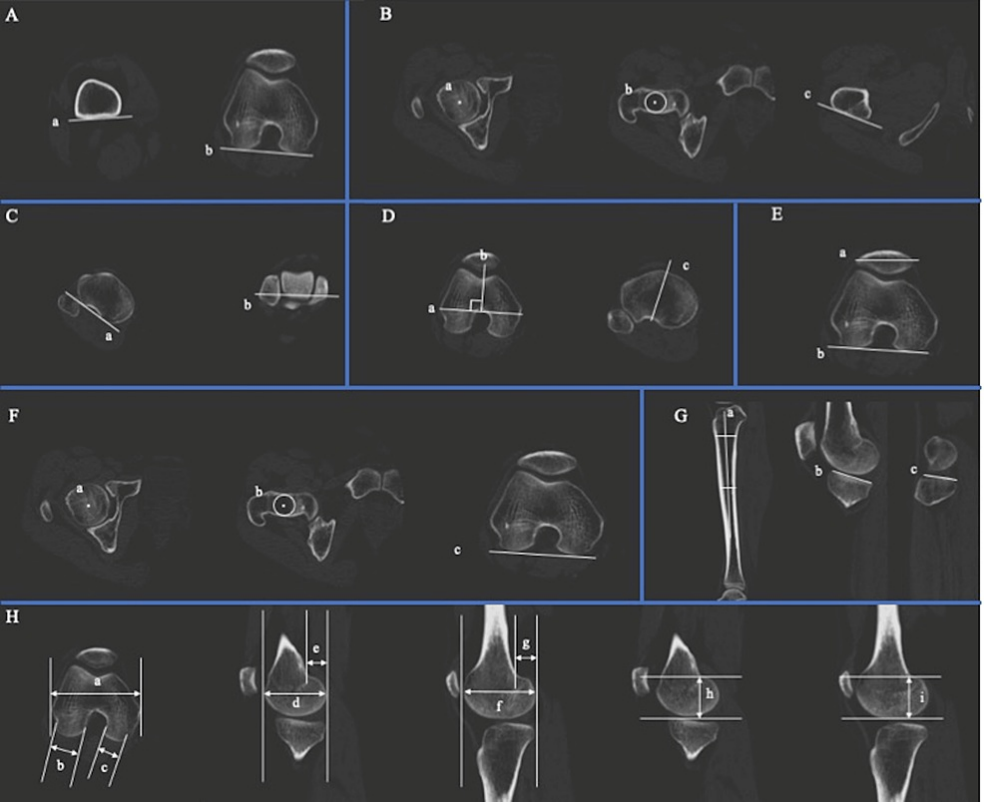
CT measurement parameters. Measured with ZedKnee® system (LEXI, Tokyo, Japan). (A) Distal femoral torsion. a: posterior tangent line of the femoral shaft; b: posterior femoral condyle tangent line. (B) Proximal femoral torsion. a: center of the femoral head; b: center of the circle tangent to the femoral neck; c: lesser trochanter tangent. (C) Tibial torsion. a: posterior tibial tangent; b: line connecting the medial and lateral malleolus of ankle. (D) Femoral tibial joint torsion. a: Surgical Epicondylar Axis (SEA); b: vertical line of the SEA; c: line connecting the PCL attachment to the medial edge of the tibial tuberosity. (E) Patellar tilt. a: patellar axis; b: posterior femoral condyle tangent line. (F) Femoral anteversion angle. a: center of the femoral head; b: center of the circle tangent to the femoral neck; c: posterior femoral condyle tangent line. (G) Medial and lateral posterior tibial slope angles. a: tibial sagittal axis; b: medial tibial plateau tangent; c: lateral tibial plateau tangent. (H) Femoral condyle measurements. a: medial-lateral width of the epicondyle; b: lateral femoral posterior condyle width; c: medial femoral posterior condyle width; d: medial femoral condyle anteroposterior diameter; e: medial femoral posterior condyle anteroposterior diameter; f: lateral femoral condyle anteroposterior diameter; g: lateral femoral posterior condyle anteroposterior diameter; h: medial femoral condyle height; i: lateral femoral condyle height.

All patients were positioned supine with their knees extended and underwent a 1 mm axial CT scan of the lower limb, encompassing the hip, knee, and ankle joints. For axis determination in the femur, the following landmarks were identified: the center of the femoral head, medial and lateral epicondyles, the most distal points of the medial and lateral condyles, and the posterior contact points of both the medial and lateral posterior condyles. In the tibia, landmarks included the proximal medial point, distal lateral point, the medial and lateral edges of the distal tibial joint surface, the PCL attachment point, and the tibial tuberosity. Based on these landmarks, femoral and tibial coordinate axes were established. For the femur, the coordinate axes included the point of origin (midpoint between the medial and lateral epicondyles), the Z-axis (line from the center of the femoral head to the origin), the X-axis (line perpendicular to the Z-axis through the point of origin, formed by the plane through the center of the femoral head and both epicondyles), and the Y-axis (line perpendicular to both the Z- and X-axes through the origin). For the tibia, the coordinate axes comprised: the origin (intersection of lines perpendicular from the PCL attachment point to the Z-axis), the Z-axis (line from the midpoint of the proximal medial/lateral point to the midpoint of the distal articular surface medial/lateral margin), the Y-axis (line perpendicular to the Z-axis from the PCL attachment point), and the X-axis (line perpendicular to both the Z- and Y-axes). Measurements were taken of distal femoral torsion, proximal femoral torsion, tibial torsion, femoral tibial joint torsion, and patellar tilt. Additionally, femoral anteversion and the medial-lateral width of the epicondyle were assessed. The widths of the medial and lateral femoral posterior condyles were also measured in the axial plane. In the sagittal plane, the following parameters were measured: medial and lateral posterior tibial slope angles, anteroposterior diameters of the medial and lateral femoral condyles, anteroposterior diameters of the medial and lateral femoral posterior condyles, and the heights of the medial and lateral femoral condyles (Figure 2) [23-27].

Distal femoral torsion was quantified by determining the angle between the posterior tangent line of the femoral shaft and the posterior femoral condyle tangent line at the upper popliteal level [23]. The proximal femoral axis, defined as a line traversing the center of the femoral head and the center of the circle tangent to the femoral neck, served as the reference for measuring proximal femoral torsion. This measurement involved calculating the angle between the proximal femoral axis and the lesser trochanter tangent [23]. Tibial torsion was measured as the angle between the posterior tibial tangent, at the PCL attachment area, and the line connecting the medial and lateral malleolus of the ankle [24]. Femoral tibial joint torsion was measured as the angle between the vertical line of the surgical epicondylar axis (SEA) and the line from the PCL attachment to the medial edge of the tibial tuberosity, which is the attachment site of the medial patellar tendon [25]. Patellar tilt was determined by the angle between the posterior femoral condyle tangent line and the patellar axis [24]. The femoral anteversion angle was calculated as the angle between the proximal femoral axis and the line tangent to the posterior femoral condyle [23]. Additionally, the medial and lateral posterior tibial slope angles were defined by the angle between the tibial sagittal axis-a straight line linking the midpoints of the cortical outer diameter 5 cm and 15 cm distal to the knee joint-and the medial and lateral tibial plateau tangents [27]. Femoral condyle measurements were performed in axial and sagittal planes, using the largest slice of the condyle, as described by Liu et al [26]. The evaluation also included the height of the medial and lateral femoral condyles, where the lateral/medial femoral condyle height was added to the evaluation items. All CT measurements were performed using the ZedKnee® system (LEXI, Tokyo, Japan) in accordance with previously published protocols [23-27].

Statistical analyses

We used Spearman’s rank correlation coefficient analysis to examine the correlation between the LDFA and each evaluated parameter. We also performed a multiple regression analysis to identify predictors of LDFA, incorporating these correlated parameters as explanatory variables. All statistical analyses were performed using IBM SPSS Statistics for Windows, Version 23 (Released 2015; IBM Corp., Armonk, New York). Statistical significance was set at p < 0.05. Post-hoc analyses revealed that our sample size of 59 achieved 80% power and maintained a 5% significance level, confirming the adequacy of our sample size for these analyses. Measurements were performed by an orthopedic specialist. The intraclass correction coefficient (ICC) for intra-observer variance across all items ranged from 0.81 to 1.00. To assess the reliability of the inter-observer variance, measurements were performed by both an orthopedic and a non-orthopedic specialist, yielding an ICC range of 0.81 to 1.00 for all parameters.

## Results

The results of age and radiographic evaluation parameters are shown in Table 1, while those for CT evaluation parameters assessed using the Zed Knee System are shown in Table 2. Tables 3, 4 display the correlations between LDFA and each evaluation parameter. Significant correlations were observed between LDFA and several parameters: age (r = 0.3999, p = 0.0002), FTA (r = 0.327, p = 0.011), HKA (r = 0.45, p < 0.01), % MA (r = -0.478, p = 0.0002) FBA (r = 0.346, p = 0.007), femoral tibial joint torsion (r = 0.299, p = 0.021), and lateral/medial femoral condyle height (r = 0.607, p < 0.01). Multiple regression analysis, which aimed to further explore parameters correlated with LDFA, found that LDFA is strongly influenced by the height of the lateral/medial femoral condyle, age, and HKA. The results of this analysis are shown in Table 5.

**Table 1:** Patient characteristics and measurement results of X-ray parameters. FTA: femoral tibial angle, HKA: hip knee ankle angle, % MA: the percentage of mechanical axis, LDFA: lateral distal femoral angle, MPTA: medial proximal tibial angle, JLCA: joint line convergence angle. Data are expressed as mean ± standard deviation or as counts.

Parameters	Values
N	59
Sex, male:female	27:32
Age (years)	71.4±8.7
FTA (°)	183.6±6.0
HKA (°)	9.7±5.8
％MA	8.1±22.0
Femoral bowing (°)	1.1±3.8
Tibial bowing (°)	1.9±2.7
LDFA (°)	89.7±2.4
MPTA (°)	84.3±3.1
JLCA (°)	4.0±2.8

**Table 2:** Measurement results of CT parameters. Data are expressed as mean ± standard deviation.

Parameters	Values
N	59
Distal femoral torsion (°)	14.4±4.6
Proximal femoral torsion (°)	19.4±8.8
Tibial torsion (°)	23.1±9.2
Femoral tibial joint torsion (°)	4.8±5.5
Medial femoral condyle anteroposterior diameter (mm)	57.2±4.2
Lateral femoral condyle anteroposterior diameter (mm)	58.2±6.2
Medial femoral posterior condyle anteroposterior diameter (mm)	19.5±2.8
Lateral femoral posterior condyle anteroposterior diameter (mm)	18.6±2.9
Medial-lateral width of epicondyle (mm)	76.8±5.4
Medial femoral posterior condyle width (mm)	24.8±2.2
Lateral femoral posterior condyle width (mm)	22.3±3.2
Medial femoral condyle height (mm)	33.7±3.8
Lateral femoral condyle height (mm)	34.5±3.5
Lateral femoral condyle height/medial femoral condyle height	1.03±0.1
Patellar tilt (°)	8.8±6.3
Femoral anteversion angle (°)	17.0±7.0
Medial posterior tibial slope angle (°)	8.8±4.3
Lateral posterior tibial slope angle (°)	8.3±3.8

**Table 3:** Correlation between LDFA and each parameter. FTA: femoral tibial angle, HKA: hip knee ankle angle, % MA: the percentage of mechanical axis, LDFA: lateral distal femoral angle, MPTA: medial proximal tibial angle, JLCA: joint line convergence angle.

Parameters	R coefficient	P value
Age	0.399	0.002
FTA	0.327	0.011
HKA	0.45	<0.01
％MA	-0.478	<0.01
Femoral bowing	0.346	0.007
Tibial bowing	0.17	0.197
MPTA	-0.197	0.135
JLCA	0.115	0.385

**Table 4:** Correlation between LDFA and CT parameters. LDFA: lateral distal femoral angle.

Parameters	R coefficient	P value
Distal femoral torsion	-0.188	0.155
Proximal femoral torsion	0.054	0.686
Tibial torsion	0.079	0.551
Femoral tibial joint torsion	0.299	0.021
Medial femoral condyle anteroposterior diameter	0.062	0.641
Lateral femoral condyle anteroposterior diameter	0.175	0.186
Medial femoral posterior condyle anteroposterior diameter	0.046	0.728
Lateral femoral posterior condyle anteroposterior diameter	0.111	0.402
Medial–lateral width of epicondyle	-0.004	0.976
Medial femoral posterior condyle width	-0.003	0.981
Lateral femoral posterior condyle width	0.083	0.534
Medial femoral condyle height	-0.103	0.436
Lateral femoral condyle height	0.247	0.059
Lateral femoral condyle height/medial femoral condyle height	0.607	<0.01
Patellar tilt	-0.107	0.418
Femoral anteversion angle	-0.068	0.608
Medial posterior tibial slope angle	-0.142	0.283
Lateral posterior tibial slope angle	-0.203	0.124

**Table 5:** Results of multiple regression analysis for parameters correlated with LDFA. HKA: hip knee ankle angle, LDFA: lateral distal femoral angle.

LDFA	coefficient	Standard error	β	T value	P value	95% CI
Lateral femoral condyle height / Medial femoral condyle height	0.496	2.949	0.431	4.236	<0.01	6.58 to 18.398
Age	0.326	0.029	0.27	2.562	0.013	0.016 to 0.131
HKA	0.3	0.046	0.261	2.234	0.023	0.015 to 0.198

## Discussion

The key finding from this study is that the LDFA is significantly influenced by the relative height of the lateral and medial femoral condyles. AKO, particularly OWHTO, is a widely recognized surgical intervention for knee OA, known for its high survival rates and favorable long-term outcomes [1-3]. A frequent cause of suboptimal clinical outcomes is undercorrection, which has been linked to the dimensions of the LDFA. Specifically, Ishimatsu et al. reported an increased risk of correction loss in patients with a preoperative LDFA exceeding 89.5 degrees in hybrid CWHTO [17]. Furthermore, Hiramatsu et al. found that a higher preoperative LDFA is associated with a steeper postoperative knee joint line oblique (KJLO), adversely impacting both radiological assessments and clinical outcomes [18]. Similarly, Takeuchi et al. reported that a large preoperative LDFA in OWHTO predisposes patients to recurrent postoperative varus deformity [19]. Although these studies have indicated that a larger LDFA associated with bigger lateral femoral condyles can disrupt planned postoperative alignment, they did not extensively explore the morphological characteristics of the LDFA. This study employs a three-dimensional analysis of the femoral condyles, demonstrating that the LDFA is predominantly determined by the height of the lateral femoral condyle compared with the medial condyle. This suggests that a greater LDFA results when the lateral condyle is relatively taller than the medial condyle. 

Hwang et al. and Matsumoto et al. evaluated the alignment of the lower extremities in Asian populations and observed that the LDFA increases with age-related femoral bowing [20,28]. Furthermore, studies have shown a decrease in the MPTA with advancing age [29]. Previous research has also documented femoral bowing and changes in MPTA associated with aging. In this study, a correlation was found between LDFA and age as well as femoral bowing. However, multiple regression analysis revealed a strong relationship between LDFA and several factors, including femoral condyle height, age, and HKA. The results indicate that the LDFA is strongly influenced by the greater height of the lateral femoral condyle relative to the medial femoral condyle. Nonetheless, it is challenging to assert that the lateral femoral condyle is inherently longer. The CPAK classification, which predicts patients’ constitutional alignment, posits that femoral bone morphology remains essentially unchanged. However, MacDessi et al. have raised concerns that this classification does not account for bone wear and bone defects [7]. Furthermore, Toyooka et al. questioned whether the high prevalence of varus deformity in Japanese individuals is due to inherent varus alignment or deformation over time [30]. Considering the observed correlation between LDFA, age, and HKA, as well as the height differences in the femoral condyles in this study, it is possible that in medial compartment knee OA, the medial femoral condyle undergoes wear, increasing the relative height of the lateral condyle, thereby influencing LDFA. Therefore, although the CPAK classification and similar frameworks are useful for predicting constitutional alignment, caution may be warranted in cases with severe varus deformities, as changes in the LDFA could result from compression of the medial femoral condyle. In addition, this study suggests that in some instances, the assumption that femoral bone morphology remains consistent with constitutional morphology, as proposed by the CPAK classification, may be inaccurate. This finding could inform future research directions.

This study possesses several limitations that warrant mention. First, the cohort comprised exclusively of Japanese patients, which restricted our ability to explore racial disparities. Second, all patients were preoperative and had relatively severe knee OA. Future studies should stratify participants based on the grade of OA to enhance understanding. Third, the retrospective nature of this study limited the ability to evaluate the degree of flexion contracture in patients. Nonetheless, the maximum flexion contracture observed was -15 degrees of extension, which we believe minimally influenced the imaging measurements. Lastly, it remains uncertain whether the height of the medial femoral condyle was altered by aging, wear, or original bone morphology. Comparative studies involving younger patients and long-term prospective research are needed in the future to address these uncertainties.

## Conclusions

The LDFA was found to be strongly influenced by the heights of the medial and lateral femoral condyles. It is suggested that the attrition of the medial femoral condyle due to aging contributes to the progression of distal femoral varus deformity, leading to an increase in the LDFA. This study indicates that the assumption of unaltered femoral bone morphology, as represented by the CPAK classification, may not hold true in certain cases, suggesting a potential area for future research.

## References

[1] Darees M, Putman S, Brosset T, Roumazeille T, Pasquier G, Migaud H (2018). Opening-wedge high tibial osteotomy performed with locking plate fixation (TomoFix) and early weight-bearing but without filling the defect. A concise follow-up note of 48 cases at 10 years' follow-up. Orthop Traumatol Surg Res.

[2] Duivenvoorden T, van Diggele P, Reijman M, Bos PK, van Egmond J, Bierma-Zeinstra SM, Verhaar JA (2017). Adverse events and survival after closing- and opening-wedge high tibial osteotomy: a comparative study of 412 patients. Knee Surg Sports Traumatol Arthrosc.

[3] Hernigou P, Medevielle D, Debeyre J, Goutallier D (1987). Proximal tibial osteotomy for osteoarthritis with varus deformity. A ten to thirteen-year follow-up study. J Bone Joint Surg Am.

[4] Evans JT, Walker RW, Evans JP, Blom AW, Sayers A, Whitehouse MR (2019). How long does a knee replacement last? A systematic review and meta-analysis of case series and national registry reports with more than 15 years of follow-up. Lancet.

[5] Skou ST, Roos EM, Laursen MB, Rathleff MS, Arendt-Nielsen L, Simonsen O, Rasmussen S (2015). A randomized, controlled trial of total knee replacement. N Engl J Med.

[6] Kim YH, Yoon SH, Park JW (2020). Does robotic-assisted TKA result in better outcome scores or long-term survivorship than conventional TKA? A randomized, controlled trial. Clin Orthop Relat Res.

[7] MacDessi SJ, Griffiths-Jones W, Harris IA, Bellemans J, Chen DB (2021). Coronal plane alignment of the knee (CPAK) classification. Bone Joint J.

[8] Lin YH, Chang FS, Chen KH, Huang KC, Su KC (2018). Mismatch between femur and tibia coronal alignment in the knee joint: classification of five lower limb types according to femoral and tibial mechanical alignment. BMC Musculoskelet Disord.

[9] Hirschmann MT, Moser LB, Amsler F, Behrend H, Leclerq V, Hess S (2019). Functional knee phenotypes: a novel classification for phenotyping the coronal lower limb alignment based on the native alignment in young non-osteoarthritic patients. Knee Surg Sports Traumatol Arthrosc.

[10] Gill GS, Joshi AB, Mills DM (1999). Total condylar knee arthroplasty. 16- to 21-year results. Clin Orthop Relat Res.

[11] Patil S, McCauley JC, Pulido P, Colwell CW Jr (2015). How do knee implants perform past the second decade? Nineteen- to 25-year followup of the Press-fit Condylar design TKA. Clin Orthop Relat Res.

[12] Howell SM, Kuznik K, Hull ML, Siston RA (2008). Results of an initial experience with custom-fit positioning total knee arthroplasty in a series of 48 patients. Orthopedics.

[13] Rivière C, Iranpour F, Auvinet E, Howell S, Vendittoli PA, Cobb J, Parratte S (2017). Alignment options for total knee arthroplasty: a systematic review. Orthop Traumatol Surg Res.

[14] Nisar S, Palan J, Rivière C, Emerton M, Pandit H (2020). Kinematic alignment in total knee arthroplasty. EFORT Open Rev.

[15] Alves P, van Rooij F, Kuratle T, Saffarini M, Miozzari H (2022). Consistent indications, targets and techniques for double-level osteotomy of the knee: a systematic review. Knee Surg Sports Traumatol Arthrosc.

[16] Pioger C, Mabrouk A, Siboni R, Jacquet C, Seil R, Ollivier M (2023). Double-level knee osteotomy accurately corrects lower limb deformity and provides satisfactory functional outcomes in bifocal (femur and tibia) valgus malaligned knees. Knee Surg Sports Traumatol Arthrosc.

[17] Ishimatsu T, Takeuchi R, Ishikawa H, Maeyama A, Osawa K, Yamamoto T (2022). Femoral morphology affects postoperative alignment of the lower extremities in hybrid closed-wedge high tibial osteotomy. Arch Orthop Trauma Surg.

[18] Hiramatsu K, Yamada Y, Nakamura N, Mitsuoka T (2022). Factors associated with postoperative knee joint line obliquity after medial open wedge high tibial osteotomy. Am J Sports Med.

[19] Takeuchi R, Ishikawa H, Miyasaka Y, Sasaki Y, Kuniya T, Tsukahara S (2014). A novel closed-wedge high tibial osteotomy procedure to treat osteoarthritis of the knee: hybrid technique and rehabilitation measures. Arthrosc Tech.

[20] Hwang D, Wook Choi M, Kim SH (2023). Age and sex differences in coronal lower extremity alignment in a healthy Asian population. Knee.

[21] Lee NK, Lee KM, Han H, Koo S, Kang SB, Chang CB (2021). Relationship between radiographic measurements and knee adduction moment using 3D gait analysis. Gait Posture.

[22] Takagawa S, Mitsugi N, Mochida Y (2020). In Asian women undergoing total knee arthroplasty, lower leg morphology in those with rheumatoid arthritis differed from those with osteoarthritis. Mod Rheumatol.

[23] Yang G, Dai Y, Dong C, Kang H, Niu J, Lin W, Wang F (2020). Distal femoral morphological dysplasia is correlated with increased femoral torsion in patients with trochlear dysplasia and patellar instability. Bone Joint J.

[24] Kaiser P, Loth F, Attal R, Kummann M, Schuster P, Riechelmann F, Schlumberger M (2020). Static patella tilt and axial engagement in knee extension are mainly influenced by knee torsion, the tibial tubercle-trochlear groove distance (TTTG), and trochlear dysplasia but not by femoral or tibial torsion. Knee Surg Sports Traumatol Arthrosc.

[25] Aglietti P, Sensi L, Cuomo P, Ciardullo A (2008). Rotational position of femoral and tibial components in TKA using the femoral transepicondylar axis. Clin Orthop Relat Res.

[26] Liu X, Ji G, Wang X, Kang H, Wang F (2017). CT-based morphological analysis of the posterior femoral condyle in patients with trochlear dysplasia. Knee.

[27] Bao L, Rong S, Shi Z, Wang J, Zhang Y (2021). Measurement of femoral posterior condylar offset and posterior tibial slope in normal knees based on 3D reconstruction. BMC Musculoskelet Disord.

[28] Matsumoto T, Hashimura M, Takayama K (2015). A radiographic analysis of alignment of the lower extremities--initiation and progression of varus-type knee osteoarthritis. Osteoarthritis Cartilage.

[29] Higano Y, Hayami T, Omori G, Koga Y, Endo K, Endo N (2016). The varus alignment and morphologic alterations of proximal tibia affect the onset of medial knee osteoarthritis in rural Japanese women: case control study from the longitudinal evaluation of Matsudai Knee Osteoarthritis Survey. J Orthop Sci.

[30] Toyooka S, Osaki Y, Masuda H (2023). Distribution of coronal plane alignment of the knee classification in patients with knee osteoarthritis in Japan. J Knee Surg.

